# Syndrome de Ballantyne compliqué d’eclampsie: à propos d’un cas et revue de la littérature

**DOI:** 10.11604/pamj.2018.30.238.15296

**Published:** 2018-07-31

**Authors:** Fatima El Mangoub, Rachid Ait Bouhou, Zakaria Idri, Jaouad Kouach, Khalid Guelzim, Driss Moussaoui Rahali

**Affiliations:** 1 Service de Gynécologie-Obstétrique, Hôpital Militaire d’Instruction Mohamed V, Rabat, Maroc

**Keywords:** Préeclampsie en miroir, Ballantyne, hydrops fœtalis, alloimmunisation materno-fœtale, Preeclampsia mirror, Ballantyne, hydrops foetalis, maternofoetal alloimmunisation

## Abstract

Le syndrome de Ballantyne ou préeclampsie en miroir est une entité clinique rare. Son ethiopathogénie reste encore mal élucidée. Son diagnostic doit être évoqué devant un syndrome œdémateux maternel associé à un état d'anasarque fœtal. Le pronostic fœtal réservé auquel peut s'associer une forte morbidité maternelle expliquent l'intérêt de poser un diagnostic précoce en identifiant son étiologie afin d'établir un traitement anténatal pouvant améliorer ainsi le pronostic materno-fœtal. Nous rapportons et discuterons à la lumière d'une revue de la littérature, le cas d'un probable syndrome de Ballantyne secondaire à un syndrome malformatif fœtal compliqué d'éclampsie chez la mère.

## Abstract (en)

Ballantyne syndrome or preeclampsia (Mirror syndrome) is a rare clinical entity. Ethiopathogenesis is still poorly elucidated. Diagnosis must be suspected in patients with maternal edematous syndrome associated with fetal anasarca. Guarded fetal prognosis can be associate with strong maternal morbidity. This highlights the role of early diagnosis based on cause detection aimed to establish an antenatal treatment that could improve maternofoetal prognosis. We report and discuss in the light of a literature review a case of a probable Ballantyne syndrome secondary to fetal malformative syndrome complicated by eclampsia in the mother.

## Introduction

Le syndrome en miroir ou pseudo préeclampsie aussi connu sous le nom de syndrome de Ballantyne est défini par l'apparition d'œdèmes maternels attribuables à l'anasarque fœtoplacentaire [1] entrant dans le cadre d'une triade associant: un hydrops fœtalis, un œdème maternel généralisé et une placentomégalie [2]. Une préeclampsie grave lui est habituellement associée [1]. Initialement décrit par John Ballantyne en 1892 [3] où il aurait été lié à l'alloimmunisation fœtomaternelle dans le système rhésus [4], ce n'est que 60 ans après que l'hydrops fœtalis non immun [2] a été décrit par Potter et depuis, d'autres associations et étiologies non immunes. Près de 90 [5], ont été rapportées [6]: l'arythmie cardiaque fœtale, la maladie d'Ebstein, les tumeurs fœtales (tératome sacrococcygien et ou placentaire), les infections virales à parvovirus B19, coxsackies virus... [3]. Cependant la pathogénie de ce syndrome reste mal connue. Nous rapportons le cas d'une préeclampsie en miroir secondaire à un hydrops foetalis non immun probablement d'origine malformative compliquée d'une éclampsie.

## Patient et observation

**Figure 1:**
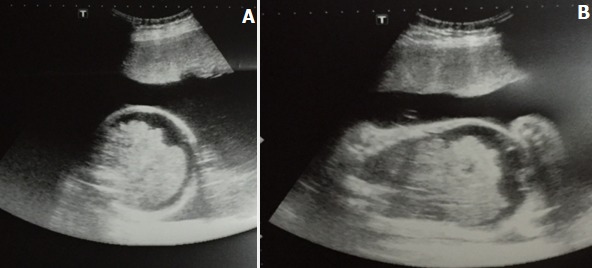
Echographie obstétricale en coupe transversale (A) et sagittale (B) montrant une anasarque fœtale avec un épanchement péritonéal, pleural et hydramnios

Primigeste de 18 ans, de groupe sanguin O Rhésus positif, sans antécédents personnels ni obstétricaux particuliers. Elle consulte aux urgences à 31 SA et 2 jours dans un tableau de préeclampsie sévère avec un syndrome œdémateux généralisé associés à des signes neurosensoriels. La grossesse était mal suivie. Elle a bénéficié de trois consultations prénatales à 14, 26 et 30 semaines d'aménorrhée et une seule échographie faite à 14 SA +4j; sans anomalie. La recherche des agglutinines irrégulières est négative, l'hémoglobinémie est à 12,7g/dL, l'hématocrite à 38,7%, les plaquettes à 250000 éléments/mm^3^ et la glycémie à 0,71g/L. Elle est immunisée contre la rubéole. Ses sérologies de la toxoplasmose, de la syphilis et de l'HIV sont négatives. Sa tension artérielle au cours du suivi était normale. L'échographie morphologique n'a pas été faite. Par ailleurs, elle a présenté une prise de poids excessive de 8kg en trois mois et demi et dont 5kg durant le dernier mois associée depuis une semaine à des œdèmes généralisés. Ce tableau clinique s'est aggravé quatre jours avant son admission par l'installation de signes neurosensoriels à type de céphalées, acouphènes et de brouillard visuel sans métrorragies ni douleurs pelviennes ou épigastriques. En effet, son examen à l'admission a trouvé une patiente en assez bon état général présentant une bouffissure du visage et d'importants œdèmes des membres inférieurs prenant le godet. Sa tension artérielle était élevée à 170/100 mmHg aux deux bras et sa bandelette urinaire était positive aux protéines (trois croix) sans nitrites. A l'examen obstétrical, les bruits du cœur fœtal étaient diminués avec contractions utérines et un mauvais relâchement inter phasique. Tandis que le toucher vaginal retrouve un col long, fermé, postérieur et un segment inferieur tendu. L'échographie obstétricale a objectivé une grossesse monofoetale évolutive en état d'anasarque fait d'un œdème sous-cutané, épanchement péritonéal et pleural ainsi qu'un hydramnios (Figure 1). Donc le diagnostic de syndrome de Ballantyne ou de préeclampsie en miroir a été posé et la décision d'extraction fœtale par voie haute a été prise devant la sévérité de la tension artérielle, les signes neurosensoriels et l'état de souffrance fœtale aigue. Diagnostic posé et après une mise en condition initiale, la patiente a bénéficié d'une extraction fœtale et fut sensibilisée au pronostic fœtal péjoratif.

**Figure 2:**
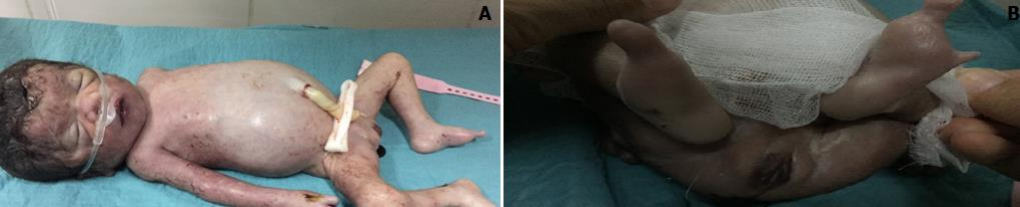
Aspect morphologique du nouveau-né prématuré montrant: une infiltration cutanée avec distension abdominale (A) et agénésie de 4 orteils (B)

**Figure 3:**
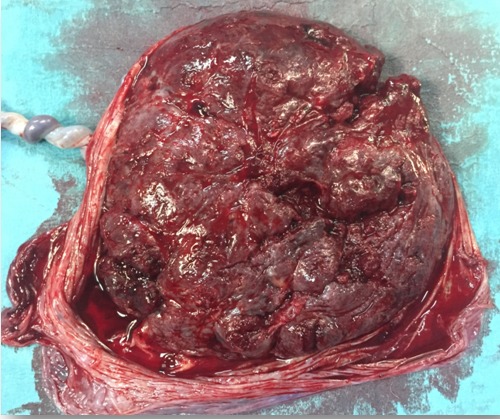
Aspect macroscopique du placenta

Une prévention d'éclampsie qui reste inhabituelle dans le syndrome en miroir, par du sulfate de magnésium ainsi qu'un bilan biologique ont été démarrés en même temps que la voie haute. La césarienne réalisée sous rachianesthésie a permis l'extraction d'un prématuré (Figure 2) pesant 1450g en état de mort apparente Apgar à 3/10 réceptionné par le pédiatre et présentant une distension abdominale, une infiltration du cuir chevelu et un syndrome malformatif fait d'agénésie de quatre orteils de chaque pied et une dextrocardie. Décédé à 30 minutes de vie malgré les mesures de réanimation néonatale ayant nécessité son intubation et sa ventilation. Les résultats du bilan fait à l'admission trouvent une hémoglobinémie à 11g/dL, une hématocrite à 27%, un taux de plaquettes à 272000 éléments/mm^3^, un taux de prothrombine à 100%, un temps de céphaline activée à 34 s/36 le témoin, une fibrinogénémie à 7g/L. Elle ne présentait pas de cytolyse hépatique, sa fonction rénale et son uricémie étaient correctes. L'exploration du délivré a montré un placenta œdémateux et hypertophique (Figure 3), adressé pour examen anatomopathologique. Lequel a montré un aspect d'hyperplacentation avec hypertrophie œdémateuse des chambres intervilleuses. L'évolution post opératoire immédiate a été marquée, et malgré l'administration du sulfate du magnésium à la seringue autopulsée, par la persistance de l'hypertension artérielle et la survenue quinze minutes après de deux crises d'éclampsie traitées par du diazepam 10mg en intraveineuse directe. Sa diurèse est restée conservée. La patiente fut transférée en unité de soins intensifs pour complément de prise en charge où les chiffres tensionels se sont corrigés sous amlodipine dix mg/jour et une dose de maintien de sulfate de magnésium fut instaurée avec contrôle régulier de son bilan biologique qui est resté normal en dehors de l'hématocrite. La patiente était transférée au service de gynécologie obstétrique après vingt quatre heures puis sortis au cinquième jour de son admission avec une consultation de cardiologie et un bilan sérologique (parvovirus B19 et cytomégalovirus). Elle a été revue à deux mois du post partum avec un monitorage de sa tension artérielle, un holter tensionnel et le résultat des sérologies virales qui sont revenus normaux.

## Discussion

Le syndrome de Ballantyne, est une entité rare en pratique clinique et souvent sous diagnostiquée. Selon Vidaeff et *al*. Vingt cas ont été rapportés dans les 46 dernières années [2]. Il complique 50% des cas d'hydrops fœtalis soit environ 1/6000 grossesses. Il survient vers la fin du deuxième ou au début du troisième trimestre [2, 4], ce qui concorde avec notre cas. Ce syndrome présente plusieurs similitudes physiopathologiques avec la prééclampsie, un tableau clinique similaire comportant constamment un syndrome œdémateux maternel « en miroir » avec l'anasarque fœtale et inconstamment une élévation de pression artérielle et des signes neurosensoriels à type de céphalées, phosphènes et acouphènes; tel est le cas de notre patiente. Sur le plan biologique, l'hémodilution est un critère quasi constant dans ce syndrome contrastant avec l'hémoconcentration habituelle dans la prééclampsie. La protéinurie décrite est faiblement augmentée [6,7]. Dans notre cas la protéinurie était positive à trois croix à la bandelette en dehors de toute infection urinaire et l'hématocrite était basse. Par ailleurs, une anémie de dilution non hémolytique est fréquente, elle peut être expliquée par l'augmentation du volume sanguin maternel et du taux élevé dans le plasma maternel de vasopressine et de facteur atrial natriurétique [3, 7]. D'autres désordres ont été décrits comme l'hyperkaliémie, l'hyponatrémie, des anomalies de la fonction rénale, une hyperuricémie [6] ou l'augmentation des transaminases [3]. Le taux de plaquettes est généralement stable. Dans notre cas, l'ionogramme, le bilan de crase, le bilan hépatique, et la fonction rénale étaient corrects. Cependant s'il existe des similitudes physiopathologiques et cliniques, les mécanismes placentaires semblent différents. Staphan et *al* [8] ont montré que le placenta dans l'hydrops fœtalis secrète de façon élevée les fms-like tyrosine kinase 1 (sFlt-1), médiateur de lésions endothéliales maternelles dans la préeclampsie sécrété de façon plus élevée dans le syndrome en miroir ce qui suggère leur physiopathologie similaire [5]. Llurba et *al* ont rapporté un syndrome en miroir secondaire à un hydrothorax fœtal bilatéral dans lequel les facteurs antiangiogéniques étaient les mêmes que ceux rencontrés dans la péeclampsie sauf que les concentrations plasmatiques en récepteurs de facteurs de croissance endothéliaux (SVEG FR-1) sont plus élevées chez les femmes avec le syndrome miroir que les femmes avec une grossesse normale mais plus basses que les patientes avec préeclampsie [5].

En effet, l'élévation du facteur (sFlt-1) a été décrite dans le syndrome miroir associé à une infection au parvovirus et au cytomégalovirus, à l'alloimmunisation foeto-maternelle et au syndrome transfuseur transfusé [8]. Toutefois, La distinction entre la prééclampsie et le syndrome miroir reste difficile. A l'inverse de la prééclampsie où on trouve une hémoconcentration ainsi qu'une diminution du volume du liquide amniotique (oligoamnios), dans le syndrome de Ballantyne, l'hématocrite maternelle est souvent diminuée par hémodilution; le liquide amniotique est souvent en excès voir un hydramnios et le fœtus est toujours en état d'anasarque. Le pronostic fœtal dans ce syndrome est péjoratif et peut avoir une morbidité maternelle grave. Ainsi la prise en charge du syndrome de Ballantyne doit être précoce et étiologique de l'hydrops foetalis, ainsi selon Ville et coll et Duthie et coll deux cas de syndrome en miroir dus à une infection au parvovirus ont été traités par transfusion in utéro ayant permis l'amélioration de la symptomatologie maternelle et la naissance d'un nouveau né à terme en bonne santé [3], ainsi qu'un cas d'arythmie fœtale corrigé par administration maternelle de flécaine a permis une amélioration des symptômes maternel et fœtaux [3]. Cependant si la cause est indéterminée, l'interruption de la grossesse permet la rémission de la symptomatologie maternelle. Dans notre cas, l'étiologie malformative est la plus probable; le pronostic fœtal étant réservé et le pronostic maternel mis en jeu, une décision d'interruption médicale de grossesse a été prise avec résolution des symptômes maternels au bout de cinq jours. Un an après, la patiente a été revue en consultation préconceptionnelle puis suivie pour sa grossesse. Sa tension artérielle, son bilan biologique sont corrects et sa protéinurie est restée négative. L'échographie morphologique est sans anomalie. Elle a bénéficié d'une césarienne programmée à 39 semaines d'aménorrhée donnant naissance à un nouveau né en bon état de santé.

## Conclusion

Le syndrome de Ballantyne est l'expression de la sévérité de la pathologie foeto-placentaire, il est de diagnostic difficile et dont l'éthiopathogénie reste encore mal expliquée; néanmoins, il est secondaire à un syndrome de rétention hydrique et d'hyperplacentation avec hypertrophie œdémateuse des chambres intervilleuses. Le pronostic fœtal est réservé pouvant mettre en jeu le pronostic maternel d'où l'intérêt de faire un diagnostic précoce afin d'envisager un traitement adapté en fonction de l'étiologie, quand cela s'avère possible, permettant la résolution de la symptomatologie maternelle et la poursuite de la grossesse. Dans le cas où la cause est incurable, l'interruption médicale de la grossesse reste la clé pour la rémission des symptômes maternels.

## Conflits d’intérêts

Les auteurs ne déclarent aucun conflit d'intérêts.

## Remerciements

Nous tenons à remercier Docteur Majdouline Boujoual qui a contribué à la correction de ce manuscrit.
